# Extended high efficacy of the combination sulphadoxine-pyrimethamine with artesunate in children with uncomplicated falciparum malaria on the Benin coast, West Africa

**DOI:** 10.1186/1475-2875-8-37

**Published:** 2009-03-03

**Authors:** Alain Nahum, Annette Erhart, Daniel Ahounou, Désiré Bonou, Chantal Van Overmeir, Joris Menten, Martin Akogbeto, Marc Coosemans, Achille Massougbodji, Umberto D'Alessandro

**Affiliations:** 1 Centre de Recherches Entomologiques de Cotonou, Cotonou, Bénin; 2 Prince Leopold Institute of Tropical Medicine, Antwerp, Belgium; 3 Laboratoire de Parasitologie, Faculté des Sciences de la Santé, Université Nationale du Bénin, Cotonou, Bénin

## Abstract

**Background:**

A study carried out in 2003–2005 in Southern Benin showed a day-28 sulphadoxine-pyrimethamine (SP) monotherapy failure rate greater than 40%, while for SP combined with artesunate (SP-AS) the failure rate was 5.3%. Such a large difference could be explained by the relatively short 28-day follow-up period, with a substantial number of recurrent infections possibly occurring after day 28. This paper reports the treatment outcome observed in the same study cohort beyond the initial 28-day follow-up.

**Methods:**

After the 28-day follow-up, children treated with either chloroquine alone (CQ), SP or SP-AS, were visited at home twice a week until day 90 after treatment. A blood sample was collected if the child had fever (axillary temperature ≥37.5°C). Total clinical failure for each treatment group was estimated by combining all the early treatment failures and late clinical failures that occurred over the whole follow-up period, i.e. from day 0 up to day 90. Pre-treatment randomly selected blood samples were genotyped for the *dhfr *gene (59) and the *dhps *gene (437 and 540) point mutations related to SP resistance.

**Results:**

The PCR-corrected clinical failure at day 90 was significantly lower in the SP-AS group (SP-AS: 2.7%, SP alone: 38.2%; CQ: 41.1%) (Log-Rank p < 0,001). The most prevalent haplotype was *dhfr *Arg-59 with the *dhps *Gly-437 mutant and the *dhps *540 wild type (85.5%). The *dhps *540 mutation could be found in only three (8.3%) samples.

**Conclusion:**

Combining artesunate to SP dramatically increased the treatment efficacy, even when extending the follow-up to day 90 post-treatment, and despite the high percentage of failures following treatment with SP alone. Such a good performance may be explained by the low prevalence of the *dhps *540 mutation, by the rapid parasite clearance with artesunate and by the level of acquired immunity.

## Background

In Benin, the current national anti-malarial drug policy was established in 2004 and selected two artemisinin-based combination therapies (ACT), artemether-lumefantrine (AL) and amodiaquine-artesunate (AQAS) as the first- and second-line treatment, respectively [1,2]. This change was needed given the high resistance to chloroquine (CQ), the first-line treatment for many years and the variable treatment efficacy of sulphadoxine-pyrimethamine (SP), the second-line treatment [3]. Indeed, though not widely used, SP treatment failure (28-day PCR uncorrected) varied from 3.3% in the north to 45.9% in Central Benin and 14.3% at the coast [3].

In Africa, SP treatment failure is strongly related to the combination of 3 mutations in the dihydrofolate reductase (*dhfr*) gene (Asn-108 + Ile-51 + Arg-59) with 2 (Gly-437 + Glu-540) in the dihydropteroate synthetase(*dhps*) gene [4,5]. The *dhfr *mutations are selected in a stepwise manner, with the Asn-108 mutation occurring first, followed by the Ile-51 and then the Arg-59 mutations. Therefore, the Arg-59 mutation is considered as a surrogate marker for the *dhfr *triple mutation [5].

The use of ACT is expected to improve the therapeutic efficacy of the treatment and also prevent the emergence and spread of *Plasmodium falciparum *drug resistance [6,7]. Though there is general consensus on the use of ACT, it is less clear which specific ACT should be chosen by a given country and on what criteria. It is often stated that the partner drug to the artemisinin derivative should also be efficacious and a lower-than-expected efficacy of an ACT has been observed where there was a substantial resistance to the partner drug [8,9]. However, in Thailand the combination mefloquine-artesunate was implemented at a time when resistance to mefloquine was extremely high [10] and this halted the progression of mefloquine resistance [11]. This may not apply to sub-Saharan Africa where the intensity of transmission is higher than in South-East Asia and could increase the probability of selecting resistant parasites to the partner drug during the period when the artemisinin derivative has already been eliminated. Furthermore, considering that a three-day dosing period with an artemisinin derivative is inadequate, it is more likely that when the partner drug is not effective, there would be a high number of therapeutic failures. Therefore, combining artesunate (AS) to sulphadoxine-pyrimethamine (SP) should only marginally improve the treatment efficacy where SP resistance is widespread. However, this is not what was observed in a study carried out in Benin in 2003, before the change of the anti-malarial drug policy, that tested both CQ and SP, the first and second-line treatment, and SP combined to AS as a potential alternative treatment. The treatment failure at day 28 with SP alone was above 40%, while that of SP combined with AS was only 5.3% [12]. Such large difference could have been dependant on the relatively short 28-day follow-up period and it was suspected that substantial recrudescence rates would occur after day 28. Therefore, follow-up was extended until day 90 after treatment and pre-treatment parasites were genotyped for molecular markers related to SP resistance. The results are reported below.

## Methods

### Study site and population

The study site and the population have already been described elsewhere [12]. Briefly, the study was carried out in three adjacent peri-urban sites in the coastal lagoon near Cotonou, where malaria is hyper-endemic [13-15]. A census of all residents was carried out before the clinical trial and a list of 6–59 month old children was produced [12]. After having obtained the parents' and/or the guardians' written informed consent, a cohort of 556 children was established by randomly selecting them from the census database.

### Study design and procedure

Between July 2003 and January 2005, children in the cohort were visited at home twice a week, had their axillary temperature checked and if it was found equal or above 37.5°C, a blood sample for microscopic examination (thick and thin blood film) and for later genotyping was collected. Similar procedures were carried out when children of the cohort attended the health facilities within the study area. Children with fever (axillary temperature ≥37.5°C), a *P. falciparum *mono-infection, with a parasite density between 1,000–200,000/μL, a PCV≥15% and without severe malaria [16], danger signs (prostration, inability to drink, recent convulsion, persistent vomiting), other concomitant illness or underlying disease were included in the clinical trial and allocated according to a predefined randomization list to either CQ (25 mg/kg over three days), SP (25 mg/kg of sulphadoxine and 1.25 mg/kg of pyrimethamine in a single dose) or SP-AS (SP: 25 mg/kg of sulphadoxine and 1.25 mg/kg of pyrimethamine in a single dose, and AS: 12 mg/kg/over three days). Children were observed for at least 1 hour after treatment; if vomiting occurred within 30 minutes, a full dose of treatment was administered, half dose if it occurred between 30 minutes and 1 hour. Allocation to treatment groups was blinded (sealed opaque envelopes) until final recruitment of the patient.

### Patients follow-up and outcome measurements

Besides the first three days of treatment (days 0, 1 and 2), children were seen at scheduled visits at days three, seven, 14, 21 and 28 [17]. The 28-day follow-up outcomes were defined according to the standard WHO classification: early treatment failure (ETF), late clinical failure (LCF), late parasitological failure (LPF) and adequate clinical and parasitological response (ACPR) [17]. All clinical failures detected beyond day 28 were defined as LCF. After having completed the standard 28-day follow-up, all children with either ACPR or LPF were visited at home twice a week up to day 90 in order to detect all possible clinical malaria attacks. During these post 28-day follow-up visits, a physician examined the child and the body temperature was checked. If the child had fever (axillary temperature ≥37.5°C), a thick blood film for detecting peripheral parasitaemia was done and a blood sample was collected on filter paper for later genotyping. Moreover, parents/legal guardians were encouraged to attend the research collaborative health centres whenever the child was sick. Quinine (24–30 mg/kg/day for seven days) was used as rescue treatment and administered to all children with ETF or LCF. Rescue treatment was also given to children with persistent vomiting following the administration of one of the study drugs. Children with an LPF were followed up and treated only if they developed clinical malaria. All treatments were given under direct observation.

### Laboratory methods

#### Parasite count and haematotological assays

Thin blood films were fixed with methanol and were stained, together with thick films, with Giemsa 10% for 10 minutes. Parasite density was determined according to the number of parasites per 200 white blood cells (WBC), and assuming a total WBC count of 8,000/μl. If gametocytes were seen, the gametocyte count was extended to 1,000 WBC. Slide reading was blinded to patients' identity and treatment allocation. Packed cell volume (PCV), measured by micro-haematocrit centrifugation, was assessed at day 0, 14 and 28.

#### Parasite genotyping

Parasite genotyping was done at day 0 and the day of recurrent parasitaemia from blood collected and dried on filter paper (Whatman filter paper grade 3). DNA was purified and genotyping done by nested PCR for variable blocks within the merozoite surface protein 1 and 2 (msp1 and msp2) as described previously [18,19]. A recrudescence was defined when at least one common band was observed for both markers in the day 0 sample and at the day of recurrent parasitaemia. A new infection was defined when no common band was observed for at least one of the two markers between day 0 and the day of recurrent parasitaemia. Patients with a recurrent infection identified as new were considered as ACPR for the PCR-corrected estimate.

#### Mutations related to SP resistance

Only children having received either SP or SP-AS were included in this analysis. Patients in the CQ group were excluded since the objective was to estimate the prevalence of molecular markers related to SP resistance and explain the high efficacy in the SP-AS arm. Patients classified as ETF were also excluded because of the previously reported lack of association between SP resistance molecular markers and ETFs [4,5].

Genotyping was done on blood samples collected before treatment at day 0 in all patients with recurrent parasitaemia and in about half of randomly selected patients having had ACPR between day 0 to 28. Considering the stepwise selection of the triple mutation, one point mutation for the *dhfr *gene (59), and two for the *dhps *gene (437 and 540) were analysed. Genotyping was carried out by mutation-specific nested polymerase chain reaction (PCR) and/or restriction digestions as described elsewhere [20]. Each *dhfr *and *dhps *codon was characterized as wild-type (no mutation present), mixed (both wild and mutant genotypes in the same infection), or pure mutant (only mutant genotypes detected).

### Statistical analysis

The follow-up time after day 28 was arbitrarily fixed at day 90 after the initial treatment. However, between day 29 and 90, blood slides were done only on children with fever so that only LCF but not LPF could be identified. Therefore, in order to make the first (day 0 to 28) and the second (day 29 to 90) follow-up periods comparable, only children experiencing either ETF or LCF between day 0 and day 90 were considered as clinical treatment failures; total clinical failure (TCF), for each treatment group was estimated by combining all the ETF and LCF that occurred over the whole follow-up period, i.e. from day 0 up to day 90. In addition, the risk of evolving towards a clinical episode was estimated for the children who during the first 28-day follow-up experienced a LPF identified as a recrudescence by PCR genotyping.

Concerning the *dhfr/dhps *mutations, infections were defined as wild type when no mutation could be detected, single mutant when only the *dhfr 59 *mutation was present, double mutant when the *dhfr 59 *mutation was present with either the *dhps 437 *or the *540 *mutations, and triple mutant when all three mutations were detected.

### Data analysis

Data were analysed with STATA version 10 (Stata Corporation, College Station, Texas, US). Descriptive statistics were used to summarize baseline values and demography data. Data not normally distributed were compared by Wilcoxon Rank sum test or Kruskal-Wallis analysis of variance. Categorical data were compared using the chi-square or the Fisher's exact test when required. The risk of clinical failure was estimated both by *Per Protocol *and Kaplan Meier survival analysis at 42, 63 and 90 day. In the Kaplan Meier survival analysis, each patient contributed to the analysis for the time s/he was followed up. When estimating TCF, data were censored for subjects who ended follow-up prior to day 90 and for new *P. falciparum *(PCR corrected) infections. In the survival analysis, clinical failure risks were described by Kaplan Meier estimates and compared between groups with a log-rank test. A p-value p < 0.05 was considered as statistically significant. Pair-wise comparisons of treatment efficacy at day 90 were made with a Cox proportional hazards model.

### Ethical approval

The study was approved by the Minister of Health of Benin, the Ethical Committee of the Faculté des Sciences de la Santé, Cotonou, Benin and by the Ethical Committee of the Institute of Tropical Medicine, Antwerp. Written informed consent was obtained from all children's parents or guardians.

## Results

### Extended follow-up until 90 days after treatment

**Figure 1:**
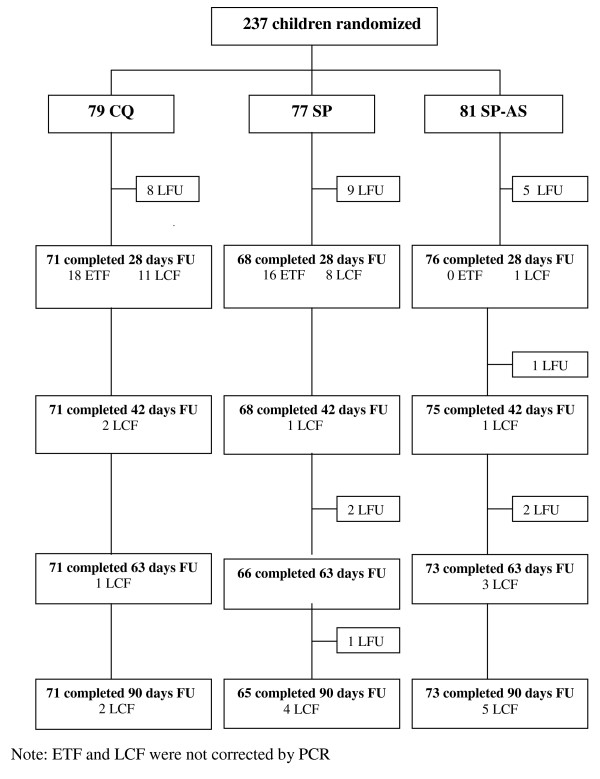
**Trial profile (90 day follow-up)**. LCF: late clinical failure. ETF: early treatment failure. CQ: Chloroquine; SP: Sulphadoxine-Pyrimethamine; AS: Artesunate. LFU: Lost to follow-up; FU: Follow-up.

**Figure 2:**
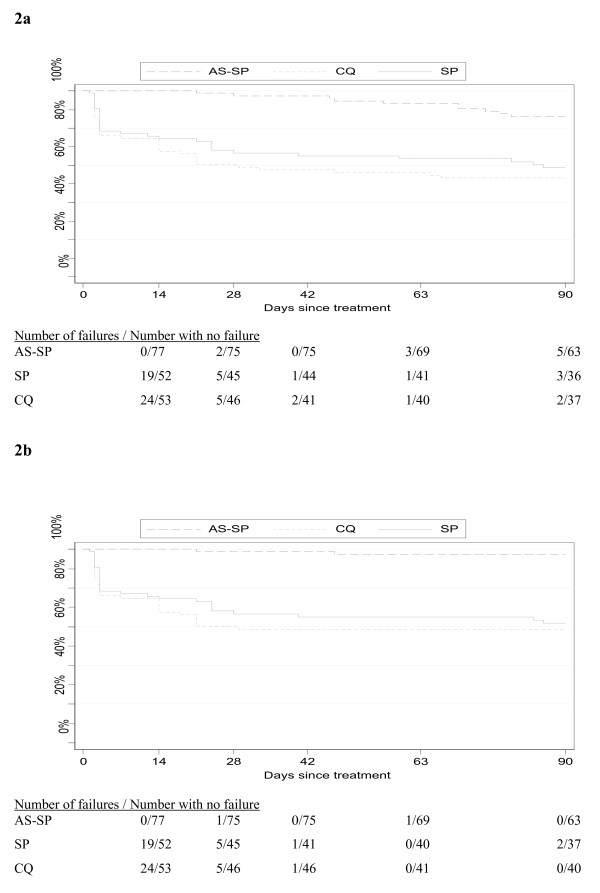
**Kaplan Meier survival curves by treatment groups and PCR correction until day 90 post-treatment**. A. PCR non-corrected. B. PCR corrected.

The baseline characteristics by treatment group and the treatment efficacy at day 28 post-treatment have already been published [12]. However, it should be noted that estimates of the treatment failure in the current analysis are different (lower) from the previous one as only ETF and LCF, but not LPF, were considered as treatment failures in order to harmonize the first 28-day follow-up and the second one from day 29 to 90. From the initial cohort of 556 children under surveillance, 237 (42.6%) children were included in the trial and randomized to one of the three study treatments. Twenty-two children (9%) did not complete the 28-day follow-up (11 withdrew consent; five moved out of study area, and six took different anti-malarials). By day 28, 54 clinical treatment failures (34 ETF and 20 LCF) received the rescue treatment. Between day 29 and 90, six additional children (three in the SP-AS and three in the SP arm) were lost to follow-up and 19 additional LCF (PCR uncorrected) were identified: four between day 29 and 42 (CQ: 2, SP: 1, and SP-AS: 1), four between day 43 and 63 (CQ: 1, and SP-AS: 3) and 11 between day 64 and 90 (CQ: 2, SP: 4, and SP-AS: 5) (Figure 1). Thus, in the *Per Protocol *analysis, TCF (PCR uncorrected) at day 90 was 13.7% (10/73) in the SP-AS, 44.6% (29/65) in the SP alone, and 47.9% (34/71) in the CQ group. After PCR correction, these rates were 2.7% (2/73), 41.5% (27/65) and 42.3% (30/71), respectively (Table 1). TCF rates estimated by Kaplan-Meier survival analysis (PCR-corrected) were similar to the *PP *analysis, i.e. SP-AS 2.7%, SP alone 38.2%, CQ 41.1% (Figure 2). Differences between monotherapy groups and SP-AS were highly significant (Log-Rank p < 0,001) and the hazard of clinical failure estimated by Cox regression both for CQ and SP were significantly higher compared to SP-AS (CQ: HR = 20.4; p < 0.001. SP alone: HR = 18.2, p < 0.001).

**Table 1:** TCF (ETF+LCF) between day 28 and day 90 by treatment group

**TCF at different day post-treatment**	**CQ**	**SP**	**SP – AS**
**Day 28: patients analysed**	**71**	**68**	**76**

TCF n (%) uncorrected:	29 (40.8)	24 (35.3)	1 (1.3)
TCF, PCR corrected:	29 (40.8)	24 (35.3)	1 (1.3)

**Day 42:**	**71**	**68**	**75**

TCF n (%) uncorrected:	31 (43.7)	25 (36.8)	2 (2.7)
TCF, PCR corrected:	30 (42.2)	25 (36.8)	1 (1.4)

**Day 63**	**71**	**66**	**73**

TCF n (%) uncorrected:	32 (45.1)	25 (37.9)	5 (6.8)
TCF, PCR corrected:	30 (42.2)	25 (37.9)	2 (2.7)

**Day 90**	**71**	**65**	**73**

TCF n (%) uncorrected:	34 (47.9)	29 (44.6)	10 (13.7)
TCF, PCR corrected:	30 (42.3)	27 (41.5)	2 (2.7)

### *Dhfr *and *dhps *mutations and therapeutic response

DNA could be amplified for the majority of blood samples (63/65) and one additional sample was not interpretable after genotyping. The prevalence of the *dhfr *59 and *dhps *437 mutations (pure or mixed) was high, both in the SP alone (*dhfr *59: 97.2%; *dhps *437: 88.9%) and in the SP-AS (*dhfr *59: 88.5%; *dhps *437: 96.2%) groups (Table 2). In contrast, the prevalence of the *dhps *540 mutation was low in the SP group (8.3%) while no mutation was found in the SP-AS group. In both treatment groups, no significant association between any individual mutation and treatment outcome was found. Even though the haplotype 59M437M540W (Arg-59 + Gly-437 + Glu-540) (85.5%) was the most represented, it was not associated to treatment failure (p = 1.0) (Table 3).

**Table 2:** Individual *dhfr *and *dhps *mutations by treatment and treatment outcome

	**Total** **SP**	**SP**		**Total** **SP-AS**	**SP-AS**		**Total**
		Treatment success	Treatment failure		Treatment success	Treatment failure	
	**N = 38**^**#**^	**N = 24**^**#**^	**N = 14**	N = 27*	N = 23*	**N = 4**	**N = 65**^#^*

***dhfr***	n (%)	n (%)	n (%)	n (%)	n (%)	n (%)	n (%)

**Arg-59**							

Wild	1 (2.8)	1 (4.5)	0 (0)	3 (11.5)	2 (9.1)	1 (25)	4 (6.5)

Mutant	25 (69.4)	16 (72.7)	9 (64.3)	18 (69.2)	15 (68.2)	3 (75)	43 (69.4)

Mixed	10 (27.8)	5 (22.7)	5 (35.7)	5 (19.2)	5 (22.7)	0 (0)	15 (24.2)

***dhps***							

**Gly-437**							

Wild	4 (11.1)	2 (9.1)	2 (14.3)	1 (3.8)	1(4.5)	0 (0)	5 (8.1)

Mutant	30 (83.3)	19 (86.4)	11 (78.6)	24 (92.3)	20 (90.9)	4 (100)	54 (87.1)

Mixed	2 (5.6)	1 (4.5)	1 (7.1)	1 (3.8)	1(4.5)	0 (0)	3 (4.8)

**Glu-540**							

Wild	33 (91.7)	21 (95.5)	12 (85.7)	26 (100)	22 (100)	4 (100)	59 (95.2)

Mutant	3 (8.3)	1 (4.5)	2 (14.3)	0 (0)	0 (0)	0 (0)	3 (4.8)

Mixed	0 (0)	0 (0)	0 (0)	0 (0)	0 (0)	0 (0)	0 (0)

# 1 not amplified and 1 not interpretable; *1 not amplified

**Table 3:** Parasite haplotypes and treatment outcome by treatment group

**Haplotype**	**SP**		**SP-AS**		**Total**
*dhfr*59*-dhps*437*-dhps*540	Success	Failure	Success	Failure	
	n (%)	n (%)	n (%)	n (%)	n (%)

M-M-W	19 (86.4)	12 (85.7)	19 (86.4)	3 (75.0)	53 (85.5)

M-W-M	1 (4.5)	2 (14.3)	0 (0)	0 (0)	3 (4.8)

M-W-W	1 (4.5)	0 (0)	1 (4.5)	0 (0)	2 (3.2)

W-M-W	1 (4.5)	0 (0)	2 (9.1)	1 (25.0)	4 (6.5)

M: Mutant; W: Wild

### Long term risk of clinical malaria among LPF and ACPR identified by day 28

By the Day 28, 38 children experienced a LPF confirmed to be a recrudescence by PCR genotyping (CQ: 25, SP: 10, and SP-AS: 3) [12]. Among these 38 LPF, 11 (28.9%) evolved towards LCF after an average of 15.4 days (95%CI: 7.9–22.9), 8 (72.7%) of them were classified as recrudescence: seven (CQ: 3 and SP: 4) before day 28 and an additional patient in the SP-AS group between day 29 and day 90 (Table 4). The 3 (27.3%) remaining LPF patients having evolved towards LCF and classified as new infections (1 by day 63 and 2 by day 90) were all in the CQ group. In the SP group, among the 10 children classified as LPF, the 4 (40%) having evolved towards clinical failure had a mean age of 26 months (95%CI: 16.9–35.1) while this was 37.7 months (95%CI: 25.7; 49.6) for those without LCF. No gametocyte was detected in LPF patients.

**Table 4:** Clinical failures (CF) by day 42, 63 and 90 post-treatment, by clinical outcome at day 28 and treatment group

**Outcome at D28°**	**Treatment group** **(N)**	**CF**					**Total CF** **D29-90**
			
			**D7-28**	**D29-42**	**D43-63**	**D64-90**	
**ACPR** (n = 123)	**SP-AS**	R	0	0	0	0	0
		
	68	N	0	0	2	5	7
	
	**CQ**	R	0	1	0	0	1
		
	18	N	0	1	0	0	1
	
	**SP**37	R	0	1	0	2	3
		
	37	N	0	0	0	1	1

**LPF new infection** (n = 7)	**SP-AS**	R	0	0	0	0	0
		
	4	N	0	1	0	0	1
	
	**CQ**	R	0	0	0	0	0
		
	2	N	0	0	0	0	0
	
	**SP**	R	0	0	0	0	0
		
	1	N	0	0	0	1	1

**LPF recrudescence** (n = 38)	**SP-AS**	R	0	0	1	0	1
		
	3	N	0	0	0	0	0
	
	**CQ**	R	3	0	0	0	3
		
	25	N	0	0	1	2	3
	
	**SP**	R	4	0	0	0	4
		
	10	N	0	0	0	0	0

°ACPR: Adequate Clinical and Parasitological Response;

LPF: Late Parasitological Failure; R = recrudescence; N = new infection; D = day).

Among the 7 patients with LPF by day 28 and classified as new infection by genotyping (in the estimation of treatment outcome considered as ACPR), 2 evolved towards LCF (SP-AS: 1, before day 42; SP: 1, before day 90) (Table 4). Among the remaining 123 ACPR patients (parasite-free by day 28), 13 (10.6%) evolved towards LCF (9 new infections and 4 recrudescences) (Table 4). Overall, the proportion of ACPR (including the LPF new infections) who evolved toward LCF was significantly lower than in the LPF group (p = 0.009). In 2 (1.5%) (SP-AS: 1, and SP: 1) of the 130 ACPR (123 parasite free and 7 new infection children by day 28) gametocytes appeared after day 28. Between day 29 and day 90, no fever was detected in the majority of children with either LPF or ACPR by day 28, so that no blood slide was taken.

## Discussion

In this area, combining AS to SP dramatically increased the treatment efficacy despite evidence of a high treatment failure at day 28 in children treated with SP alone [12]. Such a good performance of the combination could have been due to the duration of the follow-up period, not long enough to capture all failures. Indeed, it has been stated that a 42-day follow-up can capture almost all failures after treatment with anti-malarial drugs that have a terminal half-life of less than one week and that the traditional 28-day follow-up may underestimate the true failure rate by as much as 40% [21]. However, these conclusions were based on trials carried out in low transmission areas, as there were no data with a follow-up longer than 28 days in higher transmission settings [21]. In this study, the follow-up was extended up to 90 days after treatment, though failures were identified passively so that only clinical but not parasitological failures could be detected beyond the follow-up of day 28. This allowed the detection of some additional LCF; most of them by day 42 in the CQ and SP monotherapy groups, while an additional LCF was detected by day 63 in the SP-AS group. Therefore, though the 28-day follow-up misses some events, it remains a fair estimation of the efficacy of a given drug since it allowed the detection of 91.5% of all LCF (PCR corrected). However, extending the follow-up to day 42 post-treatment would give a better estimate of the drug efficacy, as 40% of the few remaining LCF (PCR corrected) after day 28 were detected before day 42.

The good efficacy of SP-AS was surprising when considering the high clinical failure rate in the SP alone group (41.5% at day 90). A three-day treatment with AS is an incomplete treatment when resistance to the partner drug is already high. However, good efficacy of the combination SP-AS, over 90% at day 28, has already been reported from some African sites with high SP resistance [22-24], while in others with similar SP resistance, the efficacy of SP-AS was not as high [25]. Moreover, effectiveness of the treatment could be as low as 63.4%, when patient's compliance is low, a problem due to the non-availability of a co-formulated treatment [24]. These results confirm that in areas where SP total clinical failure is as high as 29.4% at day 28 or 41.5% at day 90, adding AS to SP improves considerably the efficacy of the treatment. Such an improvement cannot be explained by a short follow-up unable to detect all failures. Indeed, even at day 90, the clinical failure rate in patients treated with SP-AS was as low as 2.7%.

The prevalence of the point mutations in the dihydrofolate reductase (*dhfr*) and dihydropteroate synthase (*dhps*) genes linked to SP treatment failure [5] may be a useful element to consider when trying explaining the relatively high efficacy in the SP-AS group. The *dhfr *mutations 108, 51 and 59 have been related to pyrimethamine resistance [26], whereas the *dhps *mutations 437 and 540 to sulphadoxine resistance [27,28]. The occurrence of these mutations may occur in a stepwise fashion, with selection for mutations in the *dhfr *gene probably occurring first and the *dhps *mutations following later [29]. In Uganda, the presence of the *dhps *540 mutations was a much stronger predictor of clinical treatment failure than the *dhfr *59 mutation [30]. In this study area, the *dhps *540 wild-type associated with the *dhfr *59 and *dhps *437 mutations was the most prevalent haplotype but it was not associated with SP treatment failure, possibly because of the lack of mutations in other codons, e.g. 51 in the *dhfr *gene. Treatment failure with SP associated to the *dhps *437 mutation is lower than that with the *dhps *540 mutation [30] and when AS is associated with SP such risk may be further reduced [30]. Only three samples carrying the *dhps *540 mutation with the *dhps *437 wild type were found, similar to other reports in which the occurrence of the *dhps *540 mutation without the *dhps *437 mutation was uncommon [30]. Therefore, the relatively high efficacy of SP-AS may be partly explained by the low prevalence of infections with the *dhps *540 mutation. Additional elements to be considered are the rapid effect of AS on parasite load [31] and the interaction between treatment and acquired immunity. Indeed, the reduction of parasitaemia after treatment with AS is so dramatic that SP, despite high resistance when given alone, may be able to remove the remaining parasites, helped also by the patient's acquired immunity. However, assuming that the *dhps *mutations occur in a stepwise fashion, first the *dhps *437 and then the *dhps *540 mutations, a process driven by drug pressure, and that indeed the double mutant is associated with a higher risk of failure, the efficacy of SP-AS may rapidly decrease over time if this treatment or SP alone are commonly used.

When considering only the PCR-confirmed recrudescences, only 18.4% of the LPF evolved towards a LCF within the 28-day follow-up. This proportion is much lower than that obtained in several trials carried out in sub-Saharan Africa in which more than 40% of the LPF cases evolved towards LCF within 28 days post-treatment [32]. In the study, one additional child experienced a LCF between day 29 and day 90, increasing the risk of developing clinical malaria to 21.0% (8/38). This is lower to what has been reported in Uganda where the risk of developing symptomatic malaria within 30 days was 50% [33]. Similarly, in Gabon only a small proportion of infected children remained asymptomatic for five days or more [34]. However, in the present study, more than half of these children never developed symptoms, even after a follow-up of about three months. Such difference may be explained by the age pattern of the cohort as at the end of the study the youngest children were at least 24 months old and the oldest 77 month old. Considering this is an area of intense and perennial transmission [13,14], the large majority of children probably had some degree of acquired immunity that contributed to either delaying the evolution towards clinical disease or even clearing the infection [14,35-38].

## Conclusion

Combining AS to SP dramatically increased the treatment efficacy, even when extending the follow-up to day 90 post-treatment, and despite the high percentage of failures following treatment with SP alone. Such a good performance may be explained by several factors including the low prevalence of the *dhps *540 mutation, the rapid reduction of the parasite load by AS and the interaction between acquired immunity and treatment. In 2004, Benin has chosen artemether-lumefantrine and amodiaquine-artesunate as recommended first-line treatments for uncomplicated malaria. Their deployment in peripheral health facilities has been slow though now the situation is gradually improving (A. Nahum, personal communication). Experiences from several African countries [39-42] indicate that the deployment of a new drug policy during the transition period suffers multiple constraints, including unavailability of the new treatment(s) due to stock-outs. Both SP and AS are easily available in Benin and their combination offers a good alternative whenever the two recommended anti-malaria treatments are not available [43,44].

## Competing interests

The authors declare that they have no competing interests.

## Authors' contributions

AN contributed to the study design, coordination and supervision of the field work, data entry, cleaning and analysis, and paper writing. AE contributed to the study design, protocol writing, data analysis and review of the paper. DA contributed to data management and analysis. DB contributed to data management and analysis. CVO contributed to blood sample analysis and manuscript review. JM contributed to statistical analysis. MA contributed to the study design, coordination & supervision of the field work, and paper reviewing. MC contributed to study design and paper reviewing. AM contributed to the study design, data analysis and interpretation as well as manuscript review. UDA contributed to study design, protocol writing, data analysis, interpretation and supervision, and manuscript review.
